# Biodegradable Mulching Films Based on Polycaprolactone and Its Porous Structure Construction

**DOI:** 10.3390/polym14245340

**Published:** 2022-12-07

**Authors:** Ning Yang, Li Ying, Kaiyu Li, Feng Chen, Fengyan Zhao, Zhanxiang Sun, Liangshan Feng, Jialei Liu

**Affiliations:** 1 Liaoning Academy of Agricultural Sciences, Shenyang 110161, China; 2 Key Laboratory of Water-Saving Agriculture of Northeast, Ministry of Agriculture and Rural Affairs, Shenyang 110161, China; 3 Institute of Environment and Sustainable Development in Agriculture, Chinese Academy of Agricultural Sciences, Beijing100081, China; 4 Key Laboratory of Prevention and Control of Residual Pollution in Agricultural Film, Ministry of Agriculture and Rural Affairs, Beijing 100081, China; 5 Western Agricultural Research Center, Chinese Academy of Agricultural Sciences, Changji 831100, China

**Keywords:** polycaprolactone, biodegradable, porous film, degradation property

## Abstract

Polycaprolactone (PCL) is one of the promising linear aliphatic polyesters which can be used as mulching film. Although it has suitable glass transition temperature and good biodegradability, further practical applications are restricted by the limited temperature-increasing and moisturizing properties. The rational design of the PCL structure is a good strategy to enhance the related properties. In this study, thermally-induced phase separation (TIPS) was introduced to fabricate a PCL nanoporous thin film. The introduction of a nanoporous structure on the PCL surface (np-PCL) exhibited enhanced temperature-increasing and moisturizing properties when used as mulch film. In detail, the average soil temperature of np-PCL was increased to 17.81 °C, when compared with common PCL of 17.42 °C and PBAT of 17.50 °C, and approaches to PE of 18.02 °C. In terms of water vapor transmission rate, the value for np-PCL is 637 gm^−2^day^−1^, which was much less than the common PCL of 786 and PBAT of 890 gm^−2^day^−1^. As a result, the weed biomass under the np-PCL was suppressed to be 0.35 kg m^−2^, almost half of the common PCL and PBAT. In addition, the np-PCL shows good thermal stability with an onset decomposition temperature of 295 °C. The degradation mechanism and rate of the np-PCL in different pH environments were also studied to explore the influence of nanoporous structure. This work highlights the importance of the nanoporous structure in PCL to enhance the temperature-increasing and moisturizing properties of PCL-based biodegradable mulching film.

## 1. Introduction

The application of mulching film could lead to an increase in crop yield by 20–50% because of its temperature-increasing and moisturizing properties [1]. In China, agricultural productivity has increased significantly through the application of mulching technology, mulching film plays an important role in ensuring food security in China [2]. In 2014, the number of mulching films used in China reached 1.425 million tons, covering an area of nearly 20 million hectares. To date, several mulching film materials have become important issues for agricultural production [3]. This number will increase rapidly with the increasing demand for food. Unfortunately, most of the mulching film used today is polyethylene (PE), and the widespread application of such PE-type mulching film has caused serious “white pollution” because of the residual film [4,5,6]. To solve the issue of white pollution, residual film recycling technology was applied for the prevention of white pollution at the beginning. However, this technology cannot recycle all the residual film, most of the small residual films are still in the soil which has a bad effect on further agricultural activities and underground water.

Replacing traditional polyethylene (PE) films with degradable films is an attractive way to solve the pollution of residual film in farmland, because this strategy solves the problem at the source. According to the American Society for Testing and Materials (ASTM) definition: biodegradable materials are polymer materials that can be degraded or enzymatically undergo chemical, biological, or physical changes under the action of microorganisms such as bacteria, fungi, and algae [7]. These materials can be quickly decomposed and utilized by microorganisms in nature, and the final degradation products are CO_2_ and water. The biodegradable film shows its potential of solving the white pollution problem in the future because of its environmentally friendly properties [8,9,10,11]. Although the use of biodegradable film has great potential, it is still in its infancy [12,13]. At present, it is necessary to strengthen the research on the raw materials, formulations, and production processes of biodegradable mulching film to improve its quality and reduce costs in the agricultural sectors [14,15,16]. In particular, it is necessary to develop special biodegradable mulching films for certain regions and crops to meet the requirements of agricultural production diversity.

Up to date, the degradable polymer materials mainly include diacid diol copolyester (PBS, [17] PBAT, etc.), polyhydroxyalkanoate (PHA), polycaprolactone (PCL) [18], polyhydroxybutyrate (PHB) [19], copolymer-polypropylene carbonate (PPC), and polylactic acid (PLA) [20]. Additionally, Poly(Angelica Lactone) and its copolymer with other monomers, as a new series of biodegradable polymer, was also studied in detail and they showed good thermodynamic properties and machinability [21,22,23]. Compared with other biodegradable materials, PCL is a biodegradable semi-crystalline aliphatic polyester that has only one monomer and is synthesized by ring-opening polymerization of β-caprolactone under the action of catalyst [24,25]. Its chain segment length gives it the thermodynamic characteristics suited for the application of agricultural mulching films. In addition, its glass transition temperature ranges from −60 °C to 0 °C, which is close to that of glass transition temperature PE, and its high decomposition temperature makes it much more stable than other polyesters. Herein, as a biodegradable material, PCL has wide applications including mulching film, compost bags, release carriers, microencapsulated pharmaceutical preparations, food packaging materials, surgical sutures, medical equipment, etc. [26,27,28]. PCL materials can also be mixed and manufactured with other polymer materials (including PE) to manufacture differentiated-time degradable strip films.

However, the slow crystallization rate and poor processability of PCL limits its application. Considering this, the main research concentrated mostly on blending modification with other bio-based materials [29,30,31]. While the research on its degradation performance has not received sufficient attention [32,33,34]. The introduction of a porous structure can change the surface structure of the film, which may further help the regulation of heat radiation, and is of great significance in improving the thermal insulation performance of degradable mulch film. In addition, the increase in surface area increases the contact area between water vapor and agricultural film, which is conducive to the condensation of water and is of great significance in improving the water retention performance of agricultural mulching films. In this view, np-PCL degradable film was prepared by thermally induced phase separation (TIPS). As a result, np-PCL has a less water vapor transmission rate than common PCL, which is good for preserving moisture. By mulching this kind of np-PCL film, the soil temperature was increased and the weed mass was reduced obviously when compared with the common one. As is well known, crop and environmental differences are closely related to the degradation characteristics of mulching films. For instance, soil temperatures and water influence the degradation process of biodegradable mulching film greatly [35]. Biodegradable mulching film is extremely sensitive to soil pH, light conditions and UV intensity [36,37]. Therefore, thermal stability and degradation performance of the np-PCL porous film in different solutions and at different degradation times were investigated. The degrading process was well clarified. These results are helpful for the further use of this kind of PCL film.

## 2. Materials and Methods

Chemicals used

The chemical compounds used in the present research were: polycaprolactone, industrial grade; PBAT, industrial grade; PE, industrial grade; trifluoroacetic acid, analytically pure; hydrochloric acid, analytically pure; sodium hydroxide, analytically pure; deionized water, homemade.

### 2.1. Raw Materials

Polycaprolactone was purchased from Jingtian Plastic Raw Materials Co., Ltd. (Dongguan, China) with the trade name capa-6800. It possessed a density of 1.15 g/cm^3^, a melt index of 3–10 g/10 min (160 °C/2.16 kg), number average molecular weight of about 8 × 10^4^ and an average melting temperature of 60 °C. PBAT is purchased from Guangzhou Jinfa Technology Co., Ltd. (Zhuhai, China), with the trade name D300 F20E. Its density is 1.25 g/cm^3^, melting temperature is 130 °C, average molecular weight is 2 × 10^5^, and average melting temperature is 110 °C. PE was purchased from Daqing Petrochemical (Daqing, China). Trifluoroacetic acid, analytically pure; analytical pure hydrochloric acid; sodium hydroxide, analytically pure; deionized water, homemade. Unless otherwise stated, all materials and reagents used in the experiment were purchased from Shanghai Macklin Biochemical Technology Co., Ltd. (Shanghai, China). 

### 2.2. Equipment

The equipment used in this manuscript includes: electric blast drying oven 9077A (Qingdao Mingbo Environmental Protection Technology Co., Ltd., Qingdao, China); vacuum drying oven DZF-6020 (Suzhou Jiangdong Precision Instrument Co., Ltd., Suzhou, China); scanning electron microscope JSM-5600LV (Japan Electronics Co., Ltd., Tokyo, Japan); Fourier infrared spectrometer Nicolet 6700 (Thermo Nicolet Corporation, Madison, WI, USA); thermogravimetric analyzer TG201F1 (Mettler-Toledo Group, Zurich, Switzerland); collector-type constant temperature heating magnetic stirrer DF-101 (Shenzhen Sanli Technology Co., Ltd., Shenzhen, China); electronic balance BS200S-WE2 (Germany Sartorius Group, Gottingen, Germany); magnetic heating stirrer B23-2 (Changzhou Nuoji Instrument Co., Ltd., Changzhou, China); circulating water vacuum pump SHZ-D (222) (Henan Iread Instrument Equipment Co., Ltd., Zhengzhou, China); Labthink C360-WVTR (Labthink Instruments Co., Ltd., Jinan, China); Aglient Cary 600-FTIR (Agilent Technologies Co., Ltd., Santa Clara, CA, USA). The equipment used for measuring water vapor transmittance is W3/060 water vapor transmittance measurement system of PERME brand.

### 2.3. Sample Preparation

The weighed polycaprolactone was added to trifluoroacetic acid in a certain proportion, the temperature was kept at 60 °C, and the speed was moderate so that polycaprolactone can be dissolved. The status of the dissolution process was observed. After all the raw materials were dissolved, the solution was poured into a Petri dish. The Petri dish was then placed in a drying oven to volatilize the solution and make the liquid in the Petri dish viscous. After the solution in the Petri dish was cooled, the diluent in the solution was extracted with deionized water as the extractant and pressed into a film. The prepared film material was then placed in a drying oven and dried to remove the extractant to obtain a porous film.

### 2.4. Performance Testing and Structural Characterization

The TG was used to test the thermal stability of the sample. About 1 g of sample was weighed and placed in a copper crucible at a heating rate of 10 °C/min, a scanning temperature range of 25–600 °C, and in a nitrogen atmosphere. The surface morphology of the porous film was observed by scanning electron microscopy (SEM).

Nicolet 6700 Fourier infrared spectrometer was used to analyze the chemical structure of porous film after degradation. The mass loss method was used to characterize the degradation of the film material. During the degradation of polymer materials, the products were carbon dioxide and water. The degraded polymer was dried and weighed to calculate the mass loss rate of the film. This is the direct method of observing the phenomenon and characterizing the degradation of polymer materials.

Mass loss rate:

Mn (%) = (M0 − Mn)/M0 × 100%

Mn—Mass loss percentage on day n

M0—Film mass before degradation, g

Mn—Film mass after n days of degradation, g

### 2.5. The Functional Performance of Mulching

The functional performance for mulching was carried out in May 2021, and the local rainfall was 560 mm. The width of the three studied films was set at 900 mm, and the plastic film mulching method was strip mulching. A completely randomized block design was used in the experiment. Each treatment was about 60 m^2^. The mulching crop is peanut (Baisha1016) with a planting density of 120,000 plants/667 m^2^, which is sowed and mulched with film at the end of April and harvested at the end of September.

## 3. Results and Discussion

### 3.1. Thermal Stability Analysis of Porous Film

**Figure 1:**
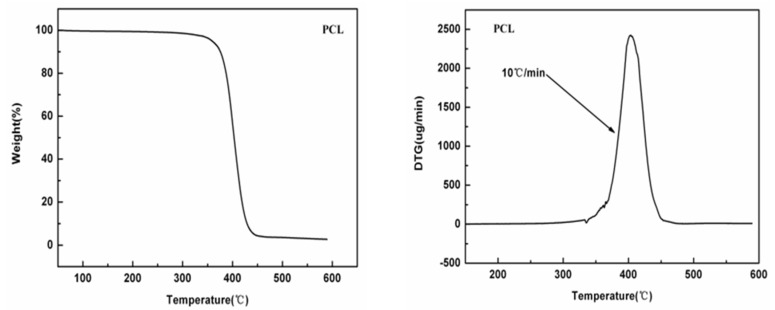
Thermogravimetric curve of the PCL porous film (**left**) and differential thermogravimetric curve of the PCL porous film (**right**).

Thermal stability is a key parameter of polymer materials, which directly determines the available processing methods and temperature resistance of such polymer materials. As the raw material of mulching film, the degradable resin must be suitable for twin-screw granulation and blow molding processes, which were accompanied by high temperature process. To investigate the thermal stability of the newly prepared np-PCL film, thermogravimetry (TG) and differential thermogravimetric (DT) were carried out. Figure 1 shows the TG (left) and DT (right) curves of the np-PCL film. As is shown, the weight of the PCL porous film was unchanged from the beginning of heating to a temperature of about 295 °C. At this stage, the weight loss of the sample was about 2%, and the main weight loss component was the adsorbed water in the material. After 295 °C, weight loss began to accelerate gradually, and 295–470 °C was the stage for rapid weight loss. At this stage, the weight loss of the sample reached about 92%, and a peak of maximum weight loss appeared at 402 °C. After that, the weight loss of the sample gradually slowed down and was unchanged from 450 °C to the end temperature of 600 °C. It can be concluded that the initial decomposition temperature of the np-PCL film is 295 °C and the maximum weight loss temperature is 402 °C, indicating that the np-PCL film has good thermal stability. Compared with the requirements of twin-screw granulation and blow molding temperature (within 200 °C), the thermal stability of this material has fully met the requirements of subsequent processing technology.

### 3.2. Analysis of the Degradation Performance of PCL Mulch Film

**Figure 2:**
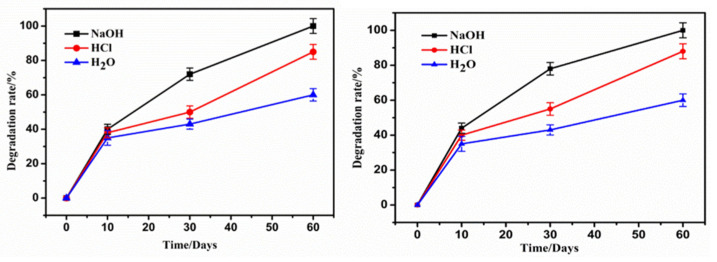
Degradation of the porous film in 0.1 mol/L (**left**) and 0.5 mol/L (**right**) solution.

Figure 2 are the time-dependent film mass loss rate for np-PCL films in three different pH solutions: NaOH, HCl, and deionized water. Figure 2 (left) and Figure 2 (right) show the results in two different concentrations of 0.1 and 0.5 mol/L, respectively. As is shown, with the increase in degradation time, the mass loss rate of the np-PCL film gradually increases, regardless of the kinds of solutions used. At the early stage (less than 10 days), the degradation rate of the np-PCL film is large, almost achieving a value of 40%. After that, the mass loss rate is relatively slow but still increases over time. This indicates that the initial stage of degradation is a process of hydrolysis which was caused by the penetrated solutions. In the later stage, the degradation originates from the degradation of polymer molecular chains which takes a long time. In comparison with the concentration of 0.1 mol/L solutions, the degree of degradation in a solution with a concentration of 0.5 mol/L is significantly slow. Under alkaline conditions, it can be found that the mass loss rate of np-PCL film is the largest, followed by HCl and H_2_O. The mass loss rate of the np-PCL film reached 100% after degradation of 60 d. Through experiments, it was found that the high or low pH environment will accelerate the degradation of np-PCL.

The PCL is a linear aliphatic polyester in which the ester bond itself is very sensitive to acidity and alkalinity. From the point of view of chemical analysis, the main mechanism of its degradation is that the ester bond on the molecular chain is hydrolyzed under acidic or alkaline conditions, which causes the molecular chain to break and produce water-soluble oligomers and monomers [26]. From the perspective of crystallization, the molecular chains in the crystalline region are arranged more closely, and the water of small molecules is not easy to penetrate. Therefore, the degradation process starts from the amorphous to the crystalline region. At the early stage of degradation, small molecules of water first penetrate the amorphous region and cause the ester bond to break, which happens easily. After most of the amorphous region has been degraded, it begins to degrade from the edge to the center of the crystalline region. However, the penetration of water molecules into the crystal zone is difficult, so the degradation rate is relatively slow [38,39]. Hydrolysis of it can produce carboxylate. Therefore, under acidic and alkaline conditions, the degradation rate of ester bonds is very fast. In an alkaline environment, HO- can react with the carboxylic acid produced by the hydrolysis reaction and is promoted to proceed in the positive direction. Therefore, the degradation rate of the PCL porous film in an alkaline solution is faster than that in an acid one, and the hydrolysis of the ester bond is not complete under acidic conditions. Additionally, the role of acid or alkali is still not entirely clear, since the decomposition of the films was carried out using dilute solutions. The precise regulation of functional period is an important indicator for the suitability evaluation of degradable plastic mulch films in crop planting. As an important index affecting the functional period of degradable plastic film, pH is firstly proposed in this article, which will be of great significance to the development of the control technology for the functional period of degradable plastic mulch film.

**Figure 3:**
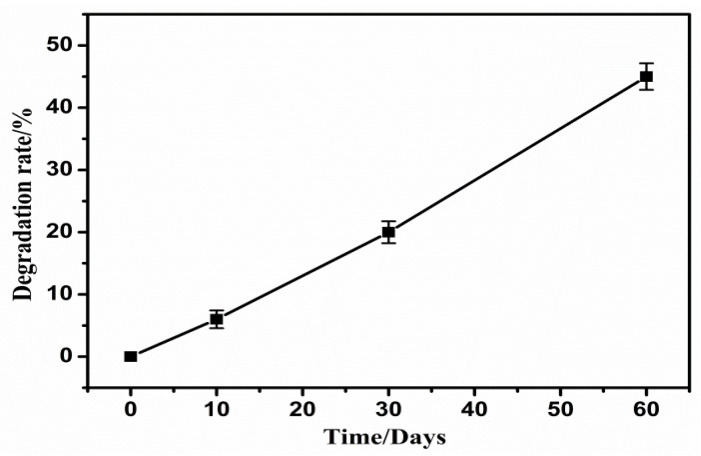
Degradation of the porous film in soil extract.

Figure 3 shows the time-dependent variation in the mass loss rate of the np-PCL film in the soil extract. It is found that the degradation rate is less than that of an alkaline solution but larger than the one in acidic and neutral solutions. This result indicates that microorganisms in the soil could be conducive to film degradation, which promotes the degradation of the porous film. However, which specific types of microbial colonies can promote degradation rate and capacity, whether these microorganisms are additive, synergistic, or inhibitory during the degradation process, and how the changes in the type and quantity during the degradation process affect the degradation process needs to be further studied. In general, 50 to 100 days are needed for 50% of biodegradable plastic film in the soil being reduced. Compared with traditional biodegradable mulching film, the degradation rate of such porous biodegradable mulching film is accelerated [40].

### 3.3. Infrared Analysis of the Porous Film

**Figure 4:**
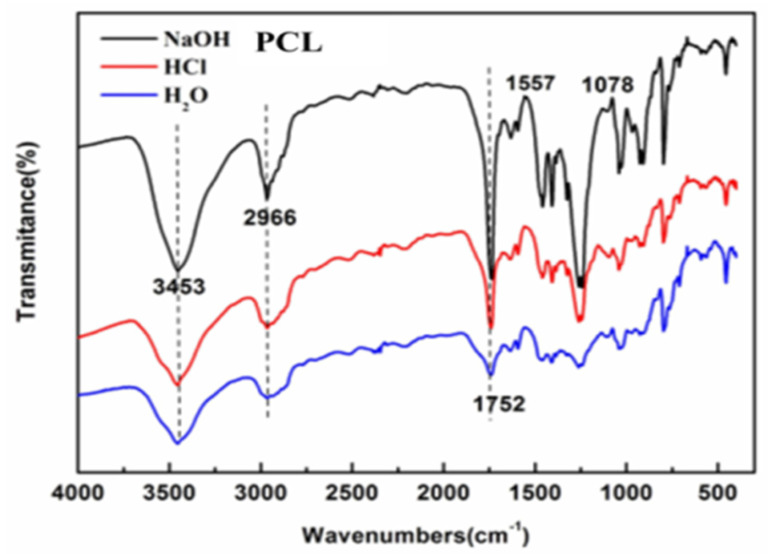
FTIR spectra of degradation samples of PCL porous film under acid, alkali and neutral conditions.

The pH has an important influence on the stability of polyester materials. By immersing the prepared films in aqueous solutions of acid, alkali, and pure water, the effect of environmental pH on the hydrolysis process of np-PCL was well studied. Here, infrared spectroscopy was carried out to monitor the surface characteristics of the immersed films and the origin infrared spectrogram of PCL refers to relevant literature [41]. As shown in Figure 4, the characteristic peaks at 3453 cm^−1^ and 2966 cm^−1^ are attributed to hydroxyl and R-COOH groups, respectively, and the peak at 1752 cm^−1^ is the C=O stretching vibration peak. These three peaks are structural characteristic peaks of PCL. Under acid and alkaline conditions, the formed peak at 1557 cm^−1^ is the characteristic peak of R-COO-, indicating that the ester bond is broken under acid and alkaline conditions. Moreover, due to the ionization of the electrolyte in the solution, a characteristic group with a negative charge is generated, which causes the ester bond to break more obviously. Under neutral conditions, no ionization occurs, so there are no charged functional groups. As a result, the rate of ester bond breaking is slow, leading to a slow degradation rate.

### 3.4. Morphological Analysis of the Porous Film

**Figure 5:**
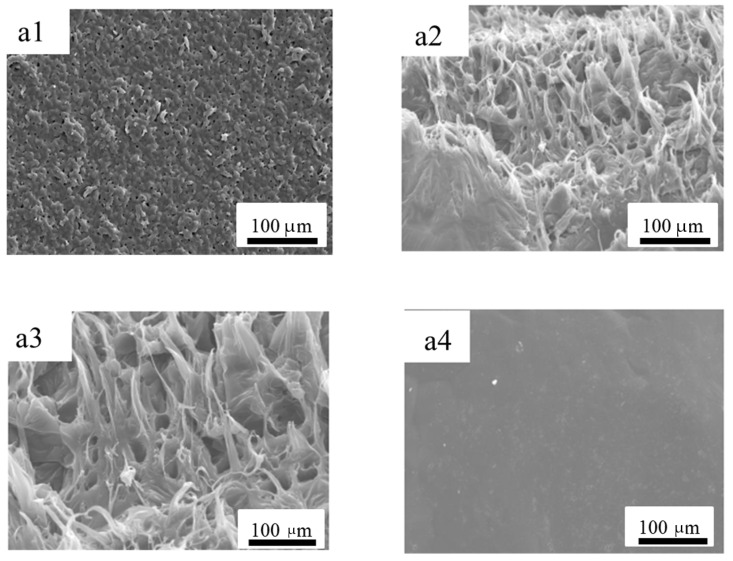
SEM images of the original PCL degradable mulching film (**a1**), and after it is degraded in NaOH (**a2**), HCl (**a3**), and deionized water (**a4**) for 30 days.

The changing of the morphology of the np-PCL can directly reflect the influence of the different pH environments. Figure 5 shows the SEM images of the surface of the np-PCL film after 30 days of degradation in NaOH, HCl, and deionized water at a concentration of 0.5 mol/L. As displayed in Figure 5(a1), the pores are evenly distributed on the surface of the prepared np-PCL film with a size of 200–400 nm. The formation of nano-scale holes is mainly caused by the extraction of volatile solvents during the drying process. Figure 5(a2) is the SEM image of the np-PCL film degraded in NaOH solution for 30 days, which obvious shows irregular large holes. The surface of the film is uneven, indicating that the nano-porous film has been partially degraded. Figure 5(a3) is the degradation diagrams of np-PCL films in acidic solutions. As is shown, the surface of the np-PCL film was eroded into irregular voids in the acidic solution, but the degradation rate was significantly lower than that in the alkaline solution. Figure 5(a4) is the degradation diagrams of np-PCL films in neutral solutions. The surface of the water-treated np-PCL film becomes flatter than a1, this change in surface structure should be attributed to the interaction between water and PCL. The hydrophilicity of polyester makes PCL absorb a certain amount of water and be swelling, which eventually leads to smaller defects. Otherwise, in an acid or alkaline environment, PCL film is degraded mainly by hydrolysis, and the defect parts are more prone to hydrolysis, so the pores will be deepened and enlarged; In the deionized water environment, the degradation process of PCL film is dominated by microbial degradation, and the contact area between microorganisms and film plays a decisive role in its degradation rate. The contact area between protrusions and microorganisms is larger, which leads to a smoother surface after degradation.

As mentioned above, the structure of the nano-porous array is very regular and can be prepared in a large area. The cost for the preparation of the nano microporous array structure is not high and the process is simple. So, such materials and processing methods are suitable for preparing functional agricultural mulching films. The porous structure of the films can afford some important properties, such as absorbability, thermal insulation performance, special optical properties, and so on [42,43]. The appearance of these functions will help to improve the comprehensive performance of agricultural mulching films. Large difference in region, different soil environments, different crop functional period needs, different light environments, and different temperatures and humidity are the key factors affecting the large-scale promotion of degradable plastic mulch films. The influence of different environments on the degradation process of degradable plastic mulch film was clarified through the change rule of the micro morphology structure in the degradation process of degradable plastic mulch film under different degradation conditions.

### 3.5. Functional Performance for Mulching

The soil temperature is measured by placing a thermometer at a depth of 5 cm below the mulching films and reading the temperature at 10:00 every day. The water vapor transmittance is measured in the laboratory, and the equipment used is the W3/060 water vapor transmittance measurement system of PERME brand.

Thermal insulation, water conservation, and weeding are the three basic functions of agricultural covering materials. To evaluate the performance of the np-PCL mulching films, common PCL, polyethylene (PE), and Poly (butyleneadipate-co-terephthalate) (PBAT) films were also prepared at the same thickness level for comparison. The functional performance including thickness, temperature of soils, water vapor transmission rate, and weed biomass of the three agricultural mulching films was listed in Table 1. Obviously, except for the thickness, all the parameters are quite different. This is reflected by the difference in chain segment structure between polyethylene and polyester. Reasonably, the soil covered by PE achieved the highest temperature of 18.02 °C, and the np-PCL one was 17.81 °C. In comparison, PBAT and common PCL film show the soil temperature of 17.50 °C and 17.42 °C, respectively. These results indicate that np-PCL film has better thermal insulation performance, which may attribute to the porous structure which can hinder the airflow on the surface of the mulching films.

It is well accepted that biodegradable polyesters such as PBAT and PCL are moisture-sensitive. Thus, the water vapor barrier property is an important parameter that should be considered when using them as mulch films. So, the water vapor transmission rate (WVTR) values of the three mulching films were measured and listed in Table 1. The WVTR value of PE was about 50 gm^−2^day^−1^. Expectedly, the WVTR value of np-PCL, common PCL, and PBAT film is one order of magnitude higher than that of PE. It is gratifying that the WVTR of np-PCL film is about 30% lower than PBAT film and 20% lower than common PCL film. Restraining weeds is also a major function of agricultural mulching films. As shown in Table 1, PE film presented the highest percentage of inhibiting weeds, followed by np-PCL film, PBAT film, and common PCL film. The improvement of the barrier property of the prepared films is related to the construction of the porous structure on the mulching film surface. The porous structure is conducive to the regulation of heat radiation and is of great significance in improving the thermal insulation performance of degradable mulch film. In addition, the increase in surface area increases the contact area between water vapor and agricultural film, which is conducive to the condensation of water and is of great significance in improving the water retention performance of agricultural mulching films. These results indicate that porous structure design is a new research direction for conserving soil moisture and suppressing weeds of fully biodegradable mulching films.

## 4. Conclusions

In this paper, new polycaprolactone (np-PCL) thin mulching films with the nanoporous structure were prepared by the thermally-induced phase separation (TIPS) method. The resulting np-PCL film has good thermal stability with an onset thermal decomposition temperature of 295 °C. On the surface of the prepared np-PCL film, nanopores are evenly distributed with a size of 200–400 nm, which may afford us excellent water permeability and thermal insulation properties. The degradation mechanism and rate of the np-PCL in different pH environments were also studied by infrared spectroscopy. It is proposed that the alkaline conditions favor the degradation of PCL-based film through the ester bonds cleavage mechanism. Consequently, the introduction of a nanoporous structure on the PCL surface (np-PCL) exhibited enhanced temperature-increasing and moisturizing properties when compared with the PBAT and common PCL mulch films. These results indicate that the introduction of the porous structure to mulching films is an important way to improve the performance of degradable mulching films. The relationship between pH and degradation rate has been clarified, which is of great significance for precise regulation of degradation period of degradable plastic film. The clarity of alkaline accelerated degradation mechanism will provide an important theoretical basis for prolonging the functional period of degradable mulch film. The WVTR of np-PCL film is about 30% lower than PBAT film and 20% lower than common PCL film, which indicated that porous structure design is a new research direction for conserving soil moisture and suppressing weeds of fully biodegradable mulching films.

## Figures and Tables

**Table 1:** Functional performance for different mulching films.

	Thickness (μm)	Temperature of Soils (°C)	Temperature Difference with PE Film (°C)	Water Vapor Transmission Rate (gm^−2^Day^−1^)	Weed Biomass (kg m^−2^)
PCL porous film	11.09	17.81	−0.21	637	0.35
PCL film	10.93	17.42	0.60	786	0.68
PBAT film	11.05	17.50	−0.52	890	0.63
PE film	10.81	18.02	0	50	0.2
